# Oncomorphic *TP*53 Mutations in Gynecologic Cancers Lose the Normal Protein:Protein Interactions with the microRNA Microprocessing Complex

**DOI:** 10.4236/jct.2014.56058

**Published:** 2014-05-16

**Authors:** Pavla Brachova, Samuel R. Mueting, Eric J. Devor, Kimberly K. Leslie

**Affiliations:** 1 Department of Obstetrics and Gynecology, University of Iowa, Iowa City, USA; 2 Molecular and Cellular Biology Program, University of Iowa, Iowa City, USA; 3 Department of Pediatrics, University of Iowa, Iowa City, USA; 4 Holden Comprehensive Cancer Center, University of Iowa, Iowa City, USA

**Keywords:** *TP*53, p53, miRNA, Drosha, Oncomorphic *TP*53, Gain of Function

## Abstract

Mutations in the tumor suppressor *TP*53 occur in almost all advanced ovarian cancers and in many advanced serous endometrial cancers. Mutations in *TP*53 can alter the function of the p53 protein, and some mutations result in a mutated protein with oncogenic activity. Previously referred to as gain of function (GOF) p53 proteins, we now term these “oncomorphic” mutations to better describe their function as oncogenes. We reviewed the data from The Cancer Genome Atlas (TCGA) and demonstrate that of the patients diagnosed with endometrial cancer that harbor *TP*53 mutations, approximately 30% of these mutations are oncomorphic. In ovarian cancer, approximately 20% are oncomorphic. The wild type (WT) p53 protein transactivates genes and micro- RNAs (miRNAs) necessary in the response to cellular stress, which turn off growth and induce apoptosis. In addition to direct transcriptional activation, WT p53 also acts through protein:protein interactions with Drosha and the miRNA processing complex to mediate rapid, enhanced processing of a subset of anti-growth miRNAs. We validated the interaction of WT p53 with the Drosha complex in the cell line UCI-107. We observed that miRNAs that inhibit the expression of oncogenes were induced. Specifically, some miRNAs were induced very rapidly over minutes, consistent with enhanced processing, while others required hours, consistent with transcriptional activation. In contrast, the most common oncomorphic *TP*53 mutations failed to interact with the Drosha complex and lost the ability to rapidly induce the miRNAs which inhibit oncogene expression. These studies highlight one mechanism underlying the oncomorphic properties of specific *TP*53 mutations: loss of the enhanced processing of anti-proliferative miRNAs.

## 1. Introduction

In the United States, endometrial cancer is the most frequent gynecologic malignancy and ovarian cancer is the most deadly. A few cancer types afflicting women, such as breast cancer or cervical cancer have prolonged survival today than that in the past 2 decades due to early detection and prevention, however; the outlook for the most deadly types of gynecologic cancers (serous ovarian and serous endometrial) has not significantly been improved [1]. Advanced serous tumors of the ovary and endometrium have similar histological and molecular appearances, clinical management, and poor patient outcomes due to high rates of chemoresistance and late diagnosis of the initial tumor. Substantially more research is needed in order to understand mechanisms involved in chemoresistance in order to achieve improved patient survival. Both serous endometrial and serous ovarian cancers exhibit extremely high rates of mutations in the tumor suppressor gene, *TP*53 (90% of serous endometrial carcinomas [2], 96% of serous ovarian carcinomas [3]). Mutations in this gene are diverse and alter the core molecular pathways involved in drug responses. Some types of *TP*53 mutations, widely termed gain of function (GOF) mutations, surprisingly convert this protein from a tumor suppressor to an oncogene. We term the resulting change an oncomorphism. While much is known about the effects of *TP*53 mutations on the oncogenic potential of the resulting mutated protein, the mechanisms governing these changes remain obscure. In this report, we describe the function of mutant p53 proteins in the microRNA (miRNA) processing pathways and the potential of these effects to impact the response to treatment.

miRNAs are short, non-coding RNA molecules comprised of 21 – 24 nucleotides. These small RNAs are involved in post-transcriptional gene regulation by binding to complementary regions in the 3' untranslated region (3'-UTR) of messenger RNAs (mRNAs) and repressing translation or inducing degradation of the target mRNA. Generally, miRNAs are transcribed by RNA polymerase II as RNA precursors with a 5'-m7G cap and 3'-poly(A) tails. The transcribed products are primary miRNAs (pri-miRs) and appear as long hairpin structures [4] [5]. Pri-miRs are processed by the RNAse III endonuclease complex, Drosha-DGCR8, to produce a shorter (~65 – 80 nucleotide) hairpin structure called a precursor miRNA (pre-miR). Pre-miRs in turn are processed by the cytoplasmic complex Dicer, which creates the mature miRNA that is able to function in the repression of target genes [6]–[8].

The miRNA processing step that occurs in the nucleus at the Drosha complex is a post-transcriptional alteration that has the potential to increase expression of mature miRNAs very rapidly. The tumor suppressor p53 is able to function in an alternative pathway, distinct from its DNA-transactivation functions, by binding to the Drosha complex and enhancing the processing of certain tumor suppressive miRNAs [9] [10]. The Drosha-mediated processing occurs through an interaction with an RNA helicase, DEAD-box RNA helicase DDX5 (p68) [11], which requires a direct protein:protein interaction with the DNA-binding domain of p53 [10]. This alternative pathway of p53 function is necessary for a quick, transcription-independent response to stress that helps cells maintain homeostasis. This alternative pathway may have important implications for cells responding to anti-cancer therapies. Because the majority of mutations in *TP*53 occur in the DNA-binding domain, it is important to understand if the mutated proteins retain the ability to alter miRNA processing. Three known mutations (C135Y, R175H, and R273H [10]) were examined and found to lose the ability to bind to the Drosha complex, but the most common p53 mutated proteins have not been examined. Therefore, we assessed the ability of the most common oncomorphic *TP*53 mutations identified in endometrial and ovarian serous tumors to bind to the Drosha complex. Our goal was to identify the impact of oncomorphic *TP*53 mutations on miRNA processing in order to predict the cellular response to stress such as chemotherapy and to understand possible mechanisms of chemoresistance.

## 2. Materials and Methods

### 2.1. Drosha Co-Immunoprecipitation (Co-IP)

Immunoprecipitations were performed using TrueBlot Beads (eBioscience). Beads were prepared according to the manufacturer’s protocol, by binding DDX5 (#9877, Cell Signaling), p53 (#2527, Cell Signaling), or IgG (#2729, Cell Signaling) control antibodies. UCI-107 cells were lysed using RIPA buffer with RNase and protease inhibitors. Immunoprecipitations were performed using the manufacturer’s protocol with the following details. Cell lysates were incubated with the TrueBlot beads for 2 hours at 4°C. After incubation, approximately ~50 ul of the supernatant was retained as input. Three 15 minute washes were performed with lysis buffer. Beads were then separated, and boiled for 5 minutes in SDS-reducing buffer.

### 2.2. Western Blot

Samples were resolved by SDS-PAGE on a 10% polyacrylamide gel, and blotted onto nitrocellulose membrane (Biological Industries, Beit Haemek, Israel). The membrane was blocked with 5% non-fat dry milk in TBST for 1 h at RT, followed by overnight incubation at 4°C with primary antibody solution, diluted 1:1000 with 5% non-fat dry milk in TBST. The membrane was washed three times in TBST and incubated for 1h at RT with horse-radish peroxidase-conjugated secondary antibody solution, diluted 1:1000 with 5% non-fat dry milk in TBST. Following three consecutive washes in TBST, the antigen antibody complex was detected by the ECL procedure (Pierce, Thermo Scientific, Rockford, IL, USA) according to manufacturer’s instructions. The proteins were visualized on X-ray film.

### 2.3. Quantitative Real Time Polymerase Chain Reaction (qPCR)

Total cellular RNA was extracted using the miRVana RNA Isolation Kit according to manufacturer’s instructions (Ambion, Life Technologies) and quantified using a NanoDrop M-1000 Spectrophotometer (Thermo Fisher Scientific, Waltham, MA). We measured the expression of miRNAs known to be associated with p53 (miR-34a, miR-34c, miR-107, miR-143, miR-145, miR-200a, miR-205), and one miRNA not reported to be associated with p53 (miR-888). For studies on mature miRNA expression, reverse transcriptions and qPCR assays were carried out using miRNA-specific RT primers and qPCR primer/probe sets (Applied Biosystems). These assays were run on an Applied Biosystems 7900 Genetic Analyzer and the resulting data were analyzed using the Applied BiosystemsStatMiner software following normalization against the RNU48 endogenous RNA control. Quantification of primary-miRNAs was performed with reverse transcription using random primers. TaqMan specific gene assays for pri-miR-34a or pri-miR-145 were normalized to GAPDH (Applied Biosystems) to detect gene expression changes. The relative mRNA levels were calculated as follows: ΔCT (sample) = CT (mRNA of interest) − CT (18S); ΔΔCT = ΔCT (post-treatment time point) − ΔCT (control); Relative expression = 2^−ΔΔCT^. Fold-change in individual mRNA levels was calculated relative to corresponding mRNA levels in control cells.

### 2.4. Cell Culture

The human ovary cancer cell line SKOV3 was purchased from American Tissue Culture Collection (ATCC, Bethesda, MD). The human ovary cancer cell line UCI-107 was a gift from Dr. Michael J. Goodheart. Cells were cultured at 37°C in a humidified 5% CO_2_ atmosphere in McCoy’s 5A medium (SKOV3) or RPMI medium (UCI-107), supplemented with 100 U/ml penicillin, 100 mg/ml streptomycin and 10% fetal calf serum.

### 2.5. Generation of Cell Models

The cell line SKOV3 has a loss of function (LOF, null) *TP*53 mutation that results in a lack of p53 protein expression. This cell line was used as a model to study the effects of the most common oncomorphic *TP*53 mutations in endometrial and ovarian tumors by stably expressing the following mutants in *TP*53: R175H, R248Q, R248Q. P72R, R248W, R273C, R273L, R273S, and Y220C as previously described [12]. Briefly, a vector containing WT p53 cDNA (Clontech) was subjected to site-directed mutagenesis (Stratagene) according to the manufacturer’s instructions. The cell line UCI-107 was used to study the effect of WT p53 on miRNA processing. WT p53 was knocked down using a shRNA plasmid vector or scrambled control (Origene).

### 2.6. Subjects

Exon sequencing data from 264 advanced serous ovarian cancer patients without a previous cancer history were downloaded from the TCGA data portal (accessed 5/06/2013). Analyses were limited to data from patients who received platinum (carboplatin, cisplatin, or oxaliplatin) and taxane (Taxotere or Paclitaxel)-based chemotherapy. Similarly, exon sequencing data was downloaded from the TCGA data portal (accessed 5/06/2013) from 71 patients diagnosed with endometrial carcinomas that had mutations in *TP*53.

### 2.7. Criteria for Designating *TP*53 Mutations

*TP*53 mutations were grouped into three categories based on functional consequence: oncomorphic, LOF, and unclassified. Oncomorphic mutations were designated based on previously published studies showing that a particular mutation causes an oncogenic phenotype. Eight *TP*53 mutations were considered oncomorphic (P151S [13] [14], Y163C [15], R175H [16]–[18], L194R [19], Y220C [20], R248Q [21], R248W [22] [23], R273C [24] [25], R273H [17] [22] [26], R273L [27], R282W [15]). LOF mutations were defined as mutations that result in the lack of protein expression. WT mutations were defined as mutations that do not alter the amino acid sequence of p53. The remaining mutations were single missense mutations, but the effect of the mutation is not fully known at this time. These mutations do not meet oncomorphic criteria, and were categorized as “unclassified” mutations. Splice mutations were also categorized into the “unclassified” category due to conflicting studies on their function [28]–[31].

### 2.8. Statistical Analysis

Each experiment was performed at least three times, and results are represented as means ± SD. Statistical analysis of data presented were performed using Graphpad Prism software. For the mRNA or miRNA qPCR data, two-tailed Student’s t-test was used in which significance was determined at p < 0.05.

## 3. Results

### 3.1. *TP*53 Mutations in Endometrial and Ovarian Serous Carcinomas

Exon sequencing data were downloaded from the TCGA data portal and mutations in *TP*53 were annotated in endometrial carcinomas as well as in advanced serous ovarian cancers (Table 1). Endometrial cancers and serous ovarian cancers had similar patterns of mutations in *TP*53, with oncomorphic mutations making up 32.4% of all mutations in endometrial cancers, and 21.2% of all mutations in ovarian cancers. Loss of function (LOF) mutations made up about 20% of all mutations in both cancer types, and unclassified *TP*53 mutations, which are point mutations not known to be oncomorphic, made up 43.7% of endometrial cancers and 59.1% of ovarian cancers. The function of these unclassified *TP*53 mutations, including their ability to bind to the Drosha complex, is unknown.

### 3.2. Development of a Cell Model to Study the Function of Wild Type p53 in miRNA Processing

**Figure 1:**
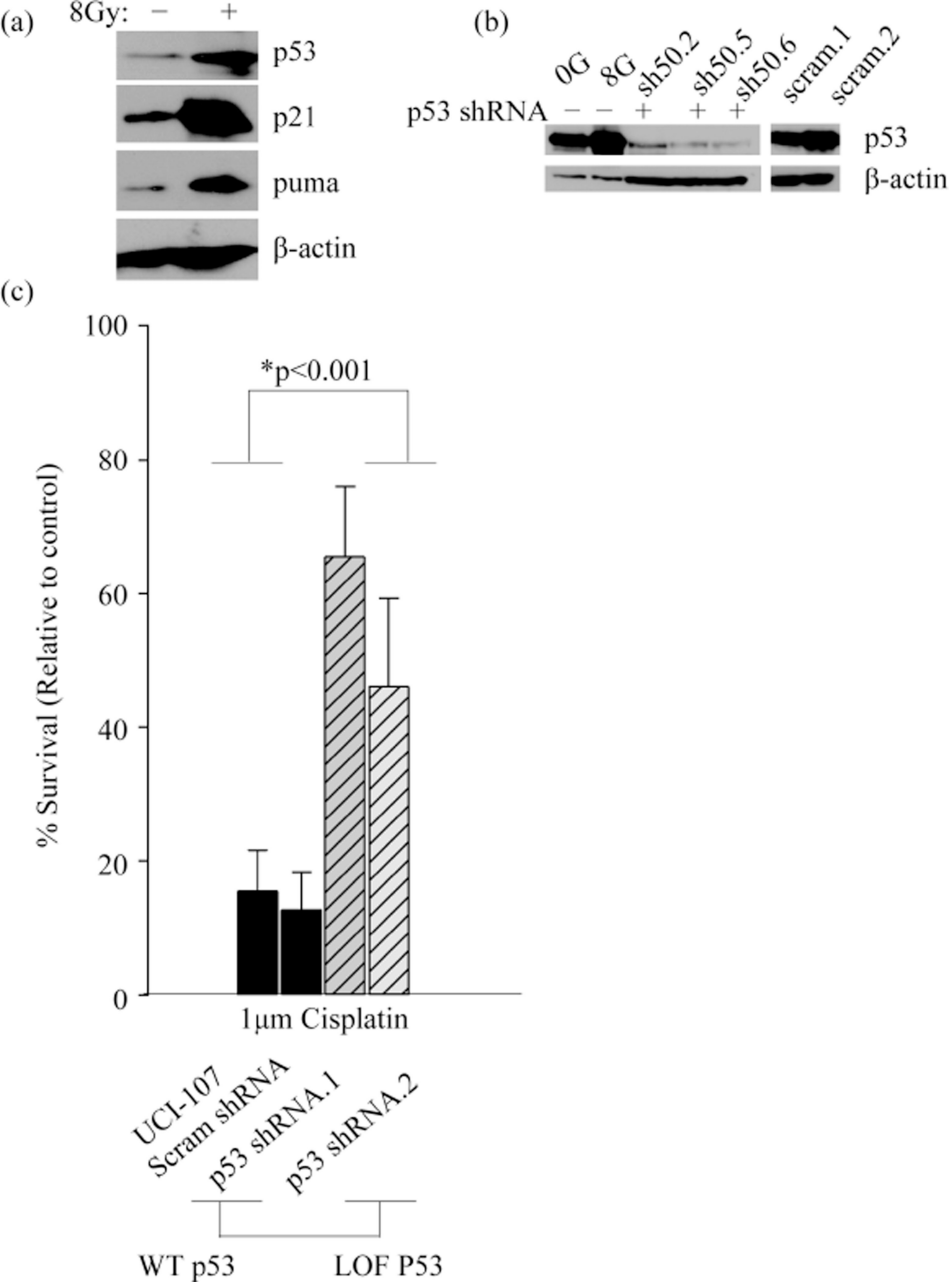
WT p53 is functional in the UCI-107 cell line. (a) The expression of p53 targets (p21 and puma) and p53 itself was measured 10 hours post 8 Gy radiation. (b) WT p53 was stably knocked down using a shRNA in UCI-107 cell lines. (c) The clonogenic potential of UCI-107 cells was examined by measuring colony formation after treatment with the chemotherapy Cisplatin.

In order to study the effect of *TP*53 mutations on miRNA processing, we first needed to establish a model to recapitulate the function of WT p53 in this process. We used the model of radiation as a classic inducer of double strand DNA breaks that activates functional, WT p53. The ovarian cancer cell line, UCI-107 has a WT *TP*53 gene that is functional based on assays that detect transcriptional activation of p53 targets p21 and puma, as well as stabilization of the p53 protein itself after activation (Figure 1(a)). To further validate the WT p53 function in this cell line, we knocked down WT p53 and generated stable cell lines (Figure 1(b)). Clones that demonstrated the highest knock-down were selected. As expected, clonogenic survival of UCI-107 cells after 1 μM cisplatin treatment was increased upon loss of WT p53 (Figure 1(c)). This further indicates that the endogenous WT p53 is functional. This was important to establish because many cancer cells without mutations in *TP*53 evolve other methods of inactivating the tumor suppressor, such as amplification of MDM2 [32].

### 3.3. WT p53 Interacts with the Drosha Complex in UCI-107 Ovarian Cancer Cell lines

**Figure 2:**
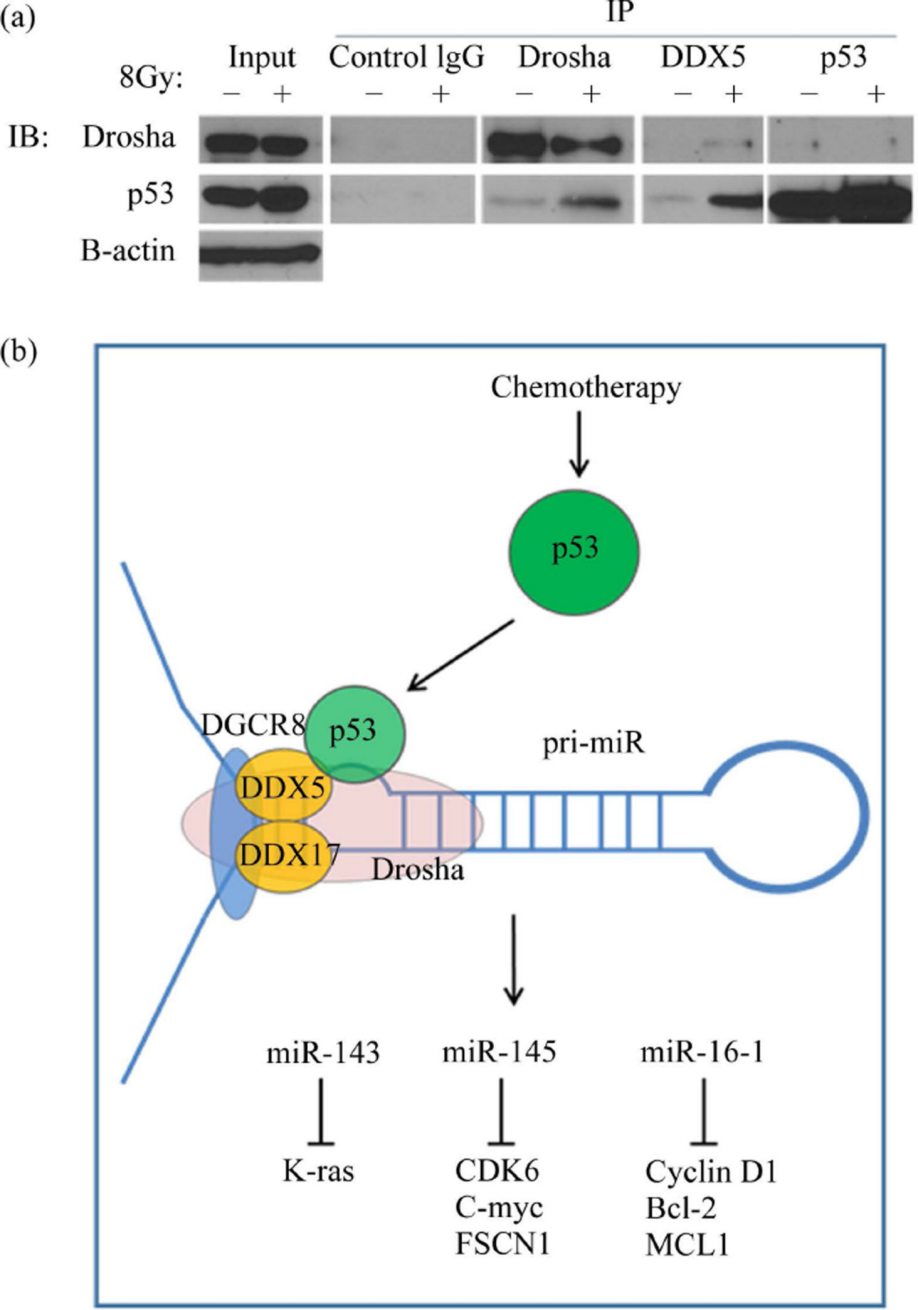
WT p53 interacts with the Drosha complex in the ovarian cancer cell line UCI-107. (a) Co-immunoprecipitation (Co-IP) experiments demonstrated an interaction between WT p53 and the Drosha complex. IPs were performed with antibodies to Drosha, DDX5, or p53. Conversely, IP of IgG did not detect any of these proteins binding to it. (b) A model representing the role of WT p53 in post-transcriptional up-regulation of tumor suppressive miRNAs. After DNA damage, p53 accumulates and is relocated to the nucleus, where it can bind to the Drosha complex and enhance the rate of miRNA processing of select miRNAs.

The ovarian carcinoma cell line UCI-107 was used to assay molecular interactions between WT p53 and Drosha. We first established that our model of p53 activation (radiation treatment) did not alter the expression of the microprocessor complex (data not shown). Therefore, any miRNA changes we detected were not due to enhanced Drosha or DDX5 expression. Co-immunoprecipitation (Co-IP) experiments demonstrated an interaction between p53, the Drosha complex, and the RNA helicase DDX5 (Figure 2(a)). This interaction was enhanced after p53 activation through radiation treatment in the DDX5 and Drosha IPs. IP of p53 was not efficient in recognizing the complex with Drosha, and was not increased by radiation. The interaction of p53 and the miRNA processing components is summarized in the model shown (Figure 2(b)).

### 3.4. miRNA Expression Changes after p53 Activation

**Figure 3:**
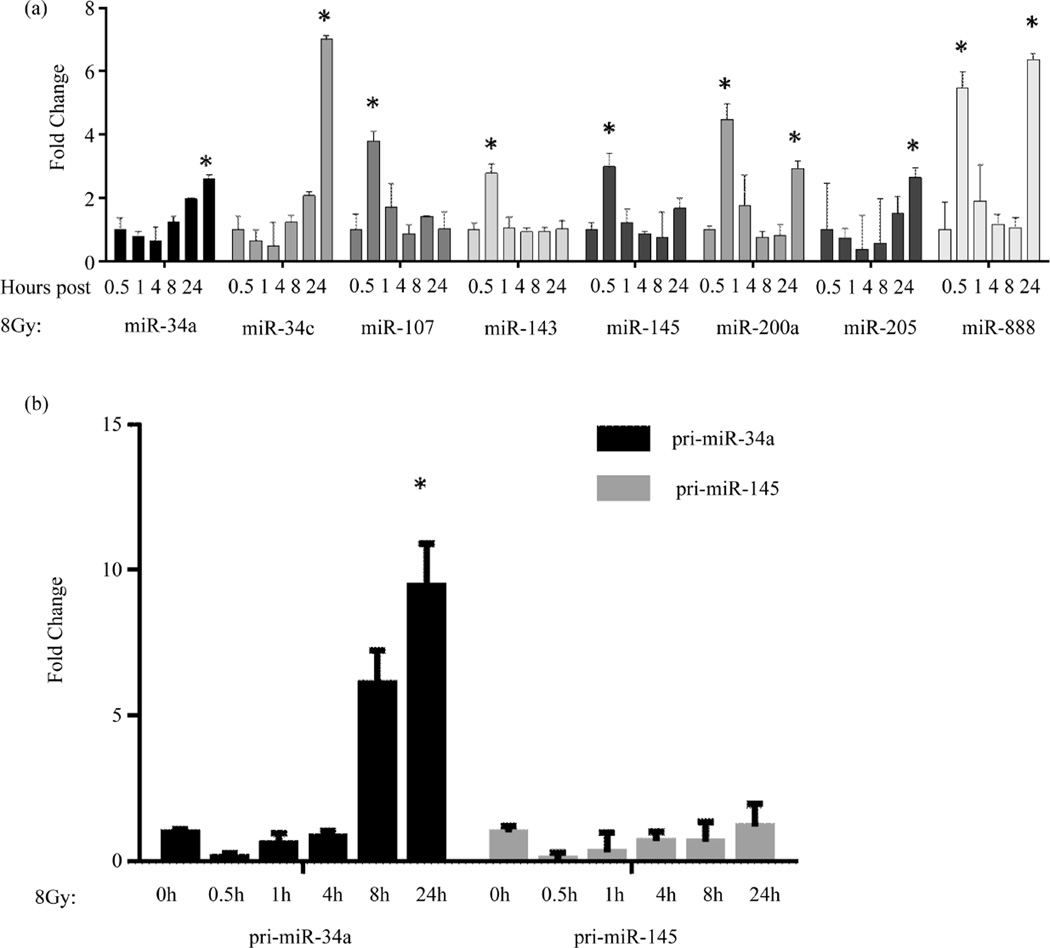
miRNA expression changes following p53 activation in UCI-107 ovarian cancer cell lines. (a) Mature miRNA expression was measured at various time points (0.5 h, 1 h, 4 h, 8 h, and 24 h) following 8G radiation treatment. miRNAs displayed two patterns of induction: short term and long term. (b) The expression of primary miRNA transcripts was measured in order to assess transcriptional activation after p53 activation.

We measured miRNA expression changes at various time points after p53 activation (Figure 3(a)). A time-course (0.5 h, 1 h, 4 h, and 8 h) was performed in order to observe miRNA expression changes after DNA-damaging radiation treatment (8Gy). Radiation treatment was used in these experiments as a means to induce p53 expression and then determine the impact of p53 on miRNA expression. After radiation treatment, there were two patterns of miRNA expression changes: short term induction and long term. Several previously reported p53-associated miRNAs were examined (miR-34a, miR-34c, miR-107 miR-143, miR-145, miR-200a, and miR-205), as well as a miRNA not previously reported to be p53-responsive (miR-888). Several of these miRNAs are direct transcriptional targets of p53 (miR-34a, miR-34c) [33], while others are known to be induced post-transcriptionally at the Drosha processing step (miR-16, miR-145) [34].

Radiation treatment demonstrated an expected increase in miRNA expression; however, there were two patterns of miRNA expression alteration: short term induction (0.5 h) and long term (8 h). These two pathways can be further discriminated by knowing the levels of pri-miR expression. In cases where a miRNA is a direct transcriptional target of p53, pri-miRs transcripts are expected to be induced; whereas, in cases where induction of the miRNAs results from p53 interaction with the microprocessing complex, pri-miRNA expression is not expected to be induced.

Several miRNAs (miR-34a, and miR-34c, miR-205) did not increase until 8 h post radiation, a time point that corresponds to transcriptional regulation. This is an example of the long term induction mechanism. In addition, miR-34a showed a corresponding increase of the pri-miR34a transcript expression at 8 h and 24 h (Figure 3(b)). Alternatively, as an example of short term induction, another set of miRNAs (miR-107, miR-143, miR-145, miR-200a, and miR-888) displayed increased miRNA expression immediately (0.5 h) after radiation therapy, indicating the more rapid post-transcriptional control. Using miR-145 as an example, we determined pri-miR levels and found that they were not induced, consistent with its regulation by direct interaction of p53 with the microprocessing complex (Figure 3(b)). One miRNA, miR-888, displayed increased expression at both the short term and the long term induction periods. It is possible that this miRNA is regulated by both transcriptional and post-transcriptional processes. The promoter contains p53 binding sites (data not shown), however, these have not yet been tested.

### 3.5. The Most Common *TP*53 Mutants Lose the Interaction with the Drosha Complex

**Figure 4:**
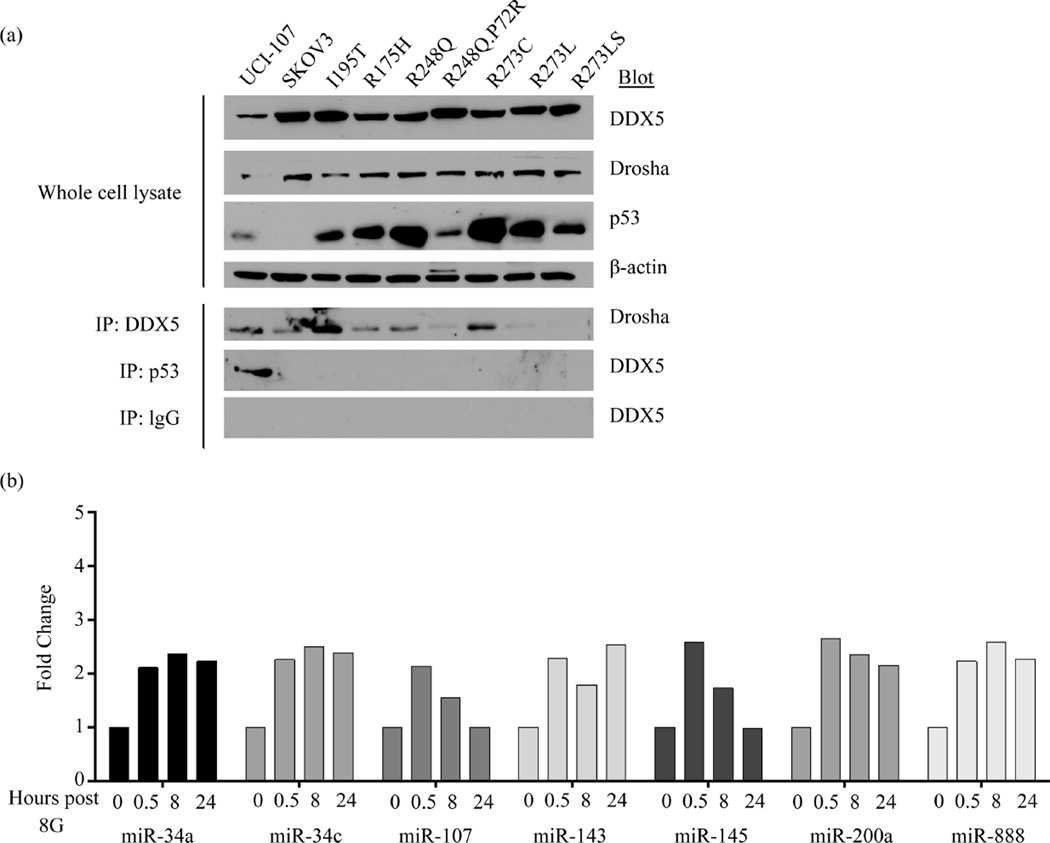
Mutant p53 proteins lose the interaction with the microprocessing Drosha complex. (a) Co-IP experiments demonstrated a loss of interaction with the Drosha complex in cell models expressing various mutated forms of p53. SKOV3 cell lines (p53 null) stably expressed p53 mutant proteins (6 oncomorphic mutations (R175H, R248Q, R248Q.P72R, R273C, R273L, and R273S) and 1 unclassified mutation (I195T). IP of DDX5 or p53 detected binding of p53 in the WT UCI-107 cell lines, but not in any other cell lines with mutant p53 proteins. (b) WT p53-responsive miRNA expression in SKOV3 cells expressing R248Q.P72R p53 mutant at a time course after radiation treatment.

WT p53 interaction with the Drosha complex is mediated through the carboxy-terminal half of the DNA binding domain of p53 [10]. Using an ovarian cancer model with various missense mutations in the DNA-binding domain, we examined the interaction of the Drosha complex and common p53 mutants (Figure 4(a)). All mutations that were examined (six oncomorphic *TP*53 mutations and one unclassified) lost the ability to bind to the Drosha complex, indicating that the important functions of miRNAs resulting from WT p53 activity are lost in the setting of common p53 mutations. In the SKOV3 cell line expressing the variant R248Q, P72R, we measured the expression of WT p53-responsive miRNAs after radiation treatment. As expected, the loss of transcriptional activity, as well as the loss of Drosha binding inhibited the induction of this set of miRNAs (Figure 4(b)). We propose that these alterations are significant for cellular function and response to chemotherapy.

## 4. Discussion

Maintenance of homeostasis is an essential process for cell survival. Cells need to respond to an ever-changing environment to maintain homeostasis, and the tumor suppressor p53 is a vital component in the response to stressors such as DNA damage, oncogene activity, or hypoxia. Similarly, because mature miRNAs, once transcribed and processed, function in a rapid manner by binding to and inactivating mRNAs, they therefore play a vital role in the rapid response to stress and the restoration of homeostasis thereafter [35]. The cooperation of p53 and miRNAs is essential in establishing a specific and swift response to stress. As such, it is predicted that mutations in the *TP*53 gene could have a drastic effect on the molecular events governing rapid restoration of homeostasis. In this report, we validate the association of WT p53 with the microprocessing complex. Suzuki *et al.* first demonstrated this relationship of p53 with the miRNA machinery [10]. We have now expanded this concept by analyzing this molecular association in the context of the most common *TP*53 mutations found in ovarian and endometrial cancers.

An in-depth analysis and characterization of ovarian and endometrial cancers performed by the TCGA demonstrated significant similarity between a subtype of high grade endometrioid and serous endometrial cancers with advanced serous ovarian cancers [2]. The TCGA analysis revealed a higher prevalence of mutations in *TP*53 than had been previously anticipated. It is now clear that the types of mutations in *TP*53 are extremely heterogeneous and can have considerably different effects on the function of the mutated protein. We divided the mutations into four groups based on the reported functions: oncomorphic (otherwise termed gain of function or GOF), loss of function (LOF or null), unclassified (single missense mutations with unknown function), and wild type (WT). *TP*53 mutations in endometrial cancers and advanced serous ovarian cancers showed a similar pattern, with oncomorphic mutations occurring in 32% and 21%, respectively. The unclassified category, constituting the majority of mutations, needs to be studied further in order to understand the dysregulated pathways that occur in the tumors that harbor them.

We examined seven common *TP*53 mutations in endometrial and ovarian carcinomas. All of these mutated p53 proteins lost the ability to associate with the Drosha complex. Since the post-transcriptional miRNA processing pathway is important in responding to stress, future experiments should address the effect of the loss of miRNA-processing efficiency on chemoresistance. The rapid expression changes of miRNAs, which occurs within minutes in response to p53 interactions with the miRNA microprocessing complex, have clinical implications. Understanding these effects is important, not only to understand mechanisms of resistance to treatment, but to predict when it occurs. miRNAs can be measured the tumors, but many are also excreted in the blood. The measurement of miRNA expression from patient serum and plasma is rapidly being developed as a clinical marker of chemoresistance or relapse [36]–[38]. In the future, we propose p53 mutational status and miRNA expression patterns as factors which should be considered and followed during therapy.

## 5. Conclusion

In conclusion, most serous endometrial and ovarian cancers harbor mutations in *TP*53. It is now clear that not all mutations in *TP*53 are equal, and studies such as this can help investigators and clinicians understand the functional significance of individual mutations. One of the main road blocks to improving survival is the high rates of chemoresistance. Our study sheds light on the function of various *TP*53 mutations and may be useful in the future development of biomarkers for treatment resistance and disease recurrence.

## Figures and Tables

**Table 1:** *TP*53 mutations in endometrial carcinomas and advanced serous ovarian cancers. Exon sequencing data downloaded from the TCGA data portal was used to annotate *TP*53 mutation types in endometrial cancers and advanced serous ovarian cancers (Brachova *et al.*, Gynecologic Oncology, submitted). LOF = loss of function or null; WT = wild type; oncomorphic = gain of function or oncogenic; unclassified = single point mutations not previously categorized as gain of function (GOF) or oncomorphic.

Endometrial Cancer			Ovarian Cancer		

*TP*53 mutation classification	Counts	Percent	*TP*53 mutation classification	Counts	%
Oncomorphic	23	32.4	Oncomorphic	56	21.2
LOF	15	21.1	LOF	50	18.9
Unclassified	31	43.7	Unclassified	156	59.1
WT	2	2.8	WT	2	0.8
Total	71	100		264	100
